# Estimation of lung cancer diagnosis and treatment costs based on a patient-level analysis in Catalonia (Spain)

**DOI:** 10.1186/s12913-015-0725-3

**Published:** 2015-02-21

**Authors:** Julieta Corral, Josep Alfons Espinàs, Francesc Cots, Laura Pareja, Judit Solà, Rebeca Font, Josep Maria Borràs

**Affiliations:** Department of Pediatrics, Obstetrics, Gynecology and Preventive Medicine, Autonomous University of Barcelona, Barcelona, Spain; Catalan Cancer Strategy, Department of Health, Generalitat de Catalunya, Barcelona, Spain; Cancer Prevention and Control [Bellvitge Biomedical Research Institute] - IDIBELL; L’Hospitalet de Llobregat, Barcelona, Spain; Parc de Salut Mar, Barcelona, Spain; Department of Clinical Sciences, University of Barcelona, L’Hospitalet de Llobregat, Barcelona, Spain

**Keywords:** Cancer, Costs, Hospital costs, Lung cancer, Non-small cell lung cancer (NSCLC), Small cell lung cancer (SCLC)

## Abstract

**Background:**

Assessing of the costs of treating disease is necessary to demonstrate cost-effectiveness and to estimate the budget impact of new interventions and therapeutic innovations. However, there are few comprehensive studies on resource use and costs associated with lung cancer patients in clinical practice in Spain or internationally. The aim of this paper was to assess the hospital cost associated with lung cancer diagnosis and treatment by histology, type of cost and stage at diagnosis in the Spanish National Health Service.

**Methods:**

A retrospective, descriptive analysis on resource use and a direct medical cost analysis were performed. Resource utilisation data were collected by means of patient files from nine teaching hospitals. From a hospital budget impact perspective, the aggregate and mean costs per patient were calculated over the first three years following diagnosis or up to death. Both aggregate and mean costs per patient were analysed by histology, stage at diagnosis and cost type.

**Results:**

A total of 232 cases of lung cancer were analysed, of which 74.1% corresponded to non-small cell lung cancer (NSCLC) and 11.2% to small cell lung cancer (SCLC); 14.7% had no cytohistologic confirmation. The mean cost per patient in NSCLC ranged from 13,218 Euros in Stage III to 16,120 Euros in Stage II. The main cost components were chemotherapy (29.5%) and surgery (22.8%). Advanced disease stages were associated with a decrease in the relative weight of surgical and inpatient care costs but an increase in chemotherapy costs. In SCLC patients, the mean cost per patient was 15,418 Euros for limited disease and 12,482 Euros for extensive disease. The main cost components were chemotherapy (36.1%) and other inpatient costs (28.7%). In both groups, the Kruskall-Wallis test did not show statistically significant differences in mean cost per patient between stages.

**Conclusions:**

This study provides the costs of lung cancer treatment based on patient file reviews, with chemotherapy and surgery accounting for the major components of costs. This cost analysis is a baseline study that will provide a useful source of information for future studies on cost-effectiveness and on the budget impact of different therapeutic innovations in Spain.

## Background

Lung cancer is the third most commonly diagnosed cancer in Spain, with approximately 32,240 newly diagnosed cases and 21,120 deaths from the disease each year. In Europe, lung cancer is also the most common cause of cancer death, with 353,460 deaths in 2012 [1].

In recent years, new drugs have been developed for lung cancer treatment, promising potential advances for patient outcomes [2]. The need to demonstrate cost-effectiveness and estimate the budget impact of new interventions and therapeutic innovations requires consideration of the costs of treating disease, including all therapeutic strategies, namely surgery, chemotherapy and radiotherapy.

However, there are very few comprehensive studies on resource use and costs associated with lung cancer patients in clinical practice in Spain or internationally. Some studies have examined the costs of lung cancer treatment, but they were based on older treatment pathways [3,4] or on simplified clinical algorithms, not on reviews of patient records [5]. Because recently introduced drugs have significantly increased the cost of the treatment [6], some studies have focused on chemotherapy treatments or on a particular phase of the disease [7-11]. A few studies have also analysed hospital costs of cancer treatment by stage at diagnosis [3,4,12,13].

It is very important to assess the cost of the entire treatment regime, not only of one therapy, in order to offer a proper perspective on the economic impact of all therapeutic strategies. The aim of this paper is to assess the hospital costs associated with lung cancer diagnosis and treatment by histology, type of cost and stage at diagnosis in the Spanish National Health Service context.

## Methods

### Patients

There are 53 public hospitals treating lung cancer patients at all stages of the disease in Catalonia; 10 hospitals (19%) have a thoracic surgery unit. As part of a global programme to assess cancer treatment in Catalonia, a clinical audit of lung cancer treatment was conducted on 1,186 incident patients from 23 hospitals that had a volume of at least 20 cases per year. For our study, a subset of 232 patients from the main patient sample was randomly selected from the 9 teaching hospitals (out of the 10 with a thoracic surgery unit) that diagnose over 50 patients per year; these hospitals also had information on their resource use available through electronic records. The sample size is composed of 197 patients diagnosed with non-small cell lung cancer (NSCLC) and 35 patients diagnosed with small cell lung cancer (SCLC), providing 80% power to detect as significant a difference of 2,000 Euros in the mean cost by stages in NSCLC patients, and 5,000 Euros in the mean cost by stages in SCLC patients. The power calculation was based on the assumption of a standard deviation of 5,250 Euros for NSCLC, and a standard deviation of 4,500 Euros for SCLC patients, using two-sided tests at a significance level of 0.05. All assumptions were based on previous studies [3,13]. Random samples were drawn proportionally to the percentage of patients diagnosed at each hospital in 2008. The Minimum Basic Data Set (MBDS) was the source of information available for the selection of cases.

Patients were categorised based on histology as either NSCLC or SCLC. When histology was unrecorded, patients fell into the category of “no cytohistologic confirmation” (NCC). NSCLC patients were classified by TNM stage at diagnosis [14] (regardless of the disease progression during treatment). SCLC patients were classified as having limited or extensive disease.

The permission to access the patient files was given by the Catalan Health Service (Department of Health of Catalonia). Approval for this study was given by the Clinical Research Ethics Committee of the Bellvitge University Hospital, since it was the referral Ethics Committee of the study coordinating institution.

### Patient-level analysis and cost estimation

A retrospective, descriptive analysis on resource use and a direct medical cost analysis were carried out. Resource utilisation data were collected by means of hospital administrative databases and patient files. From a hospital budget impact perspective, the aggregate and mean costs per patient were assessed in the three years following diagnosis or up to death. Both aggregate and mean costs per patient were analysed by stage at diagnosis and cost type. Mean cost per patient was also analysed by sex, age group and survival at one year from diagnosis. The categories of cost considered included (a) diagnosis (diagnostic tests, ambulatory and emergency visits, inpatient nights), (b) surgery (operating room and inpatient nights), (c) chemotherapy (day hospital and cytotoxic drugs), (d) radiotherapy (number of fractions), (e) other inpatient care (not related to diagnosis, surgery, chemotherapy or radiotherapy treatments for lung cancer, e.g., hospital admissions related to COPD or other comorbidities and palliative care), and (f) continuing care (ambulatory and emergency visits, tests, scans, biopsies, etc., made in the continuing phase). The mean costs of surgical interventions as well as chemotherapy and radiotherapy treatments were also assessed by histology and stage at diagnosis.

To assess the cost of the episodes of care in the period considered, unit costs from 2008 were obtained from two sources: from one hospital (Hospital del Mar) with a detailed analytical accounting system implemented over 10 years ago, and from the Spanish Network of Hospital Costs Database (RECH), which has data from 13 hospitals in Spain and it has been registered and accredited by the Spanish Ministry of Health [15,16]. The year of diagnosis was considered the baseline year (Year 0) with costs from later calendar years being discounted at 3%.

### Statistical analysis

A descriptive summary of the patients’ characteristics was formulated, reporting age, gender, TNM stage at diagnosis and treatment received. Kaplan-Maier estimates of survival time were stratified by histology and stage at diagnosis. A descriptive analysis was done to evaluate aggregate and mean cost per patient. Mann–Whitney tests were used to identify mean cost differences between sex (male/female), age group (<65 years/≥65 years) and survival at one year from diagnosis (alive one year/dead one year); and Kruskal-Wallis tests were performed to identify differences between stages. In addition, a descriptive analysis of mean costs of surgical, chemotherapy and radiotherapy treatments were carried out. All calculations were made using SPSS for Windows 21.0.

## Results

### Patient characteristics and survival

A total of 232 cases of lung cancer were analysed, of which 172 (74.1%) were NSCLC and 26 (11.2%) were SCLC; 34 (14.7%) had no cytohistologic confirmation. Table 1 describes age, gender, staging distribution of patients at diagnosis and treatments received.

**Table 1:** **Patient characteristics**

	**NSCLC**	**SCLC**	**NCC**	**Total**
**N**	**172**	**26**	**34**	**232**
**Age (years),** mean	65.4	67.3	72.7	66.7
**Gender (%)**				
Male	84.9	88.5	73.5	83.6
Female	15.1	11.5	26.5	16.4
**TNM Stage (%)**				
I	22.7	-	5.9	-
II	6.4	-	-	-
III	20.9	-	11.8	-
IV	41.3	-	47.1	-
Limited disease	-	26.9	-	-
Extensive disease	-	61.5	-	-
Unknown	8.7	11.5	35.3	-
**Surgery (%)**	**34.9**	**-**	**11.8**	**27.6**
Partial lobectomy	3.3	-	25.0	4.6
Lobectomy	59.	-	75.0	60.0
Bilobectomy	11.5	-	-	10.8
Pneumonectomy	9.8	-	-	9.2
Exploratory thoracotomy	8.2	-	-	7.7
Others	8.2	-	-	7.7
**Chemotherapy (%)**	**53.5**	**80.8**	**20.6**	**51.7**
* Intention*				
Neoadjuvant	3.0	-	-	2.2
Adjuvant	5.9	-	-	4.5
Radical	8.8	25.9	-	11.2
Palliative	45.2	41.9	46.2	44.7
Unknown	37.0	32.3	53.8	37.4
* Treatment scheme*				
Cisplatin/Carboplatin + Docetaxel	5.9	-	7.7	5.0
Cisplatin/Carboplatin + Etoposide	6.7	67.7	-	16.8
Cisplatin/Carboplatin + Gemcitabine	23.7	-	7.7	18.4
Cisplatin/Carboplatin + Paclitaxel	6.7	3.2	15.4	6.7
Cisplatin/Carboplatin + Pemetrexed	5.9	-	7.7	5.0
Cisplatin/Carboplatin + Vinorelbine	16.3	-	7.7	12.8
Docetaxel	4.4	-	7.7	3.9
Erlotinib	11.9	3.2	15.4	10.6
Pemetrexed	8.1	-	15.4	7.3
Others	10.3	25.8	15.4	13.4
**Radiotherapy (%)**	**35.5**	**69.2**	**8.8**	**35.3**
Radical	31.0	20.8	0.0	26.8
Palliative	53.5	50.0	83.3	54.4
Unknown	15.5	29.2	16.7	18.8

The median survival time was not estimable in Stages I, II (Table 2). The mean survival time decreased in NSCLC patients from Stage I to IV ranging from 36.3 to 10.7 months and in SCLC patients between limited disease and extensive disease from 17.0 to 9.4 months.

**Table 2:** **Survival time by histology and stage at diagnosis**

	**N**	**Deaths N (%)**	**Survival time (months) Median**	**Survival time (months) Mean [sd]**	**Follow-up time (months) Mean [sd]**
**NSCLC**					
Stage I	39	6 (15.4)	-	36.3 [2.2]	21.2 [13.1]
Stage II	11	3 (27.3)	-	32.1 [3.9]	21.5 [13.3]
Stage III	36	17 (47.2)	16.0	18.9 [2.8]	11.9 [11.4]
Stage IV	71	44 (62.0)	4.9	10.7 [1.9]	5.6 [7.9]
Unknown	15	5 (33.3)	-	20.1 [4.5]	10.1 [12.1]
**Total**	**172**	**75 (43.6)**	**18.0**	**22.8 [1.6]**	**11.9 [12.5]**
**SCLC**					
Limited disease	7	4 (57.1)	10.9	17.0 [3.6]	12.6 [7.1]
Extensive disease	16	13 (81.3)	8.6	9.4 [2.5]	8.4 [9.1]
Unknown	3	2 (66.7)	1.1	1.3 [0.4]	1.3 [1.0]
**Total**	**26**	**19 (73.1)**	**8.9**	**11.8 [2.6]**	**8.7 [8.5]**
**NCC**					
Stage I	2	0 (0.0)	-	-	36.3 [0.2]
Stage II	-	-	-	-	-
Stage III	4	2 (50.0)	-	-	11.9 [13.6]
Stage IV	16	11 (68.8)	-	-	4.8 [8.7]
Unknown	12	3 (25.0)	-	-	1.8 [2.1]
**Total**	**34**	**16 (47.1)**	**-**	**-**	**6.4 [10.9]**

NSCLC: non-small cell lung cancer.

SCLC: small cell lung cancer.

NCC: No cytohistologic confirmation.

sd: standard deviation.

### Cost estimation

Table 3 shows the aggregate costs of treatment by histology, stage at diagnosis, and cost category over the three years following diagnosis or up to death. In NSCLC cases, the aggregate hospital cost of lung cancer treatment ranged from 177,320 Euros at Stage II to 1,068,133 Euros at Stage IV. The aggregated cost in Stage IV was the highest, representing 41.9% of the total cost. In SCLC cases, the aggregate cost for limited disease was assessed at 107,929 Euros, whereas the cost for extensive disease was assessed as 199,707 Euros, representing 61.6% of the total cost.

**Table 3:** **Aggregate hospital costs of lung cancer diagnosis and treatment by stage at diagnosis and cost type (Euros, 2008)**

	**Diagnosis**	**Surgery**	**Chemotherapy**	**Radiotherapy**	**Other inpatient care**	**Continuing care**	**Total cost**
**NSCLC (sum, %)**							
Stage I	28,573	306,114	27,280	12,755	102,652	42,153	**519,526**
	5.5%	58.9%	5.3%	2.5%	19.8%	8.1%	100.0%
Stage II	16,106	81,416	27,624	6,538	32,288	13,348	**177,320**
	9.1%	45.9%	15.6%	3.7%	18.2%	7.5%	100.0%
Stage III	119,463	71,598	98,958	25,969	128,901	30,957	**475,846**
	25.1%	15.0%	20.8%	5.5%	27.1%	6.5%	100.0%
Stage IV	287,012	22,863	488,195	23,205	227,928	18,930	**1,068,133**
	26.9%	2.1%	45.7%	2.2%	21.3%	1.8%	100.0%
Unknown	71,792	98,615	111,030	900	15,828	9,670	**307,835**
	23.3%	32.0%	36.1%	0.3%	5.1%	3.1%	100.0%
**Total (N = 172)**	**522,946**	**580,605**	**753,087**	**69,367**	**507,597**	**115,058**	**2,548,661**
	20.5%	22.8%	29.5%	2.7%	19.9%	4.5%	100.0%
**SCLC (sum, %)**							
Limited disease	11,822	-	33,959	11,699	42,933	7,516	**107,929**
	11.0%		31.5%	10.8%	39.8%	7.0%	100.0%
Extensive disease	45,011	-	83,077	10,928	48,729	11,962	**199,707**
	22.5%		41.6%	5.5%	24.4%	6.0%	100.0%
Unknown	14,584	-	67	-	1,266	526	**16,444**
	88.7%		0.4%		7.7%	3.2%	100.0%
**Total (N = 26)**	**71,417**	**-**	**117,103**	**22,627**	**92,928**	**20,005**	**324,079**
	22.0%		36.1%	7.0%	28.7%	6.2%	100.0%
**NCC (sum, %)**							
Stage I	2,195	16,844	-	-	83,428	701	**103,168**
	2.1%	16.3%			80.9%	0.7%	100.0%
Stage II	-	-	-	-	-	-	**-**
Stage III	8,122	-	7,400	-	5,875	1,880	**23,277**
	34.9%		31.8%		25.2%	8.1%	100.0%
Stage IV	58,543	7,835	81,106	4,747	32,462	2,973	**187,665**
	31.2%	4.2%	43.2%	2.5%	17.3%	1.6%	100.0%
Unknown	62,873	10,660	2,335	-	20,794	1,698	**98,360**
	63.9%	10.8%	2.4%		21.1%	1.7%	100.0%
**Total (N = 34)**	**131,734**	**35,339**	**90,841**	**4,747**	**142,559**	**7,252**	**412,472**
	31.9%	8.6%	22.0%	1.2%	34.6%	1.8%	100.0%

NSCLC: non-small cell lung cancer.

SCLC: small cell lung cancer.

NCC: no cytohistologic confirmation.

Table 4 shows the mean cost per patient of lung cancer treatment by histology, sex, age group, stage at diagnosis, survival at one year from diagnosis and cost type. Mean cost per patient in NSCLC ranged from 13,218 Euros in Stage III to 16,120 Euros in Stage II. The main cost components in global mean cost per patient in NSCLC were chemotherapy (29.5%) and surgery (22.8%). The surgical and inpatient care cost represented 58.9% in Stage I, while in Stage II it was 45.9%, decreasing its relative weight as disease stage progressed. In addition, the relative chemotherapy cost increased in more advanced stages from 5.2% in Stage I to 45.7% in Stage IV. The relative cost of other inpatient care not related to lung cancer, including comorbid diseases, was stable between stages.

**Table 4:** **Mean hospital costs of lung cancer diagnosis and treatment per patient by sex, age group, stage at diagnosis, survival at one year from diagnosis and cost type (Euros, 2008)**

	**Diagnosis**		**Surgery**		**Chemotherapy**		**Radiotherapy**		**Other inpatient care**		**Continuing care**		**Total mean cost**		**p-value**
**NSCLC** (mean, [sd], %)															
**Male**	2,998	[3,264]	3,272	[4,838]	3,261	[8,354]	432	[785]	2,982	[4,805]	678	[910]	13,624	[10,649]	0.072
(N = 146)	22.0%		24.0%		23.9%		3.2%		21.9%		5.0%		100.0%		
**Female**	3,277	[3,089]	3,955	[5,542]	10,654	[19,139]	242	[595]	2,777	[4,738]	616	[979]	21,521	[19,367]	
(N = 26)	15.2%		18.4%		49.5%		1.1%		12.9%		2.9%		100%		
**<65 years**	2,880	[3,058]	3,436	[5,338]	4,890	[10,060]	403	[740]	2,981	[5,078]	679	[906]	15,270	[12,600]	0.716
(N = 76)	18.9%		22.5%		32.0%		2.6%		19.5%		4.4%		100.0%		
**≥65 years**	3,167	[3,372]	3,327	[4,628]	3,973	[11,634]	403	[781]	2,928	[4,561]	661	[932]	14,460	[12,674]	
(N = 96)	21.9%		23.0%		27.5%		2.8%		20.2%		4.6%		100.0%		
**Stage I**	733	[551]	7,849	[4,515]	699	[2,179]	327	[745]	2,632	[5,285]	1,081	[864]	13,321	[8,316]	0.289
(N = 39)	5.5%		58.9%		5.2%		2.5%		19.8%		8.1%		100.0%		
**Stage II**	1,464	[1,131]	7,401	[4,898]	2,511	[4,887]	594	[811]	2,935	[4,142]	1,213	[1,333]	16,120	[7,632]	
(N = 11)	9.1%		45.9%		15.6%		3.7%		18.2%		7.5%		100.0%		
**Stage III**	3,318	[2,576]	1,989	[4,159]	2,749	[6,452]	721	[978]	3,581	[5,051]	860	[1,235]	13,218	[10,240]	
(N = 36)	25.1%		15.0%		20.8%		5.5%		27.1%		6.5%		100.0%		
**Stage IV**	4,042	[3,299]	322	[1,610]	6,876	[13,283]	327	[661]	3,210	[4,853]	263	[416]	15,044	[14,338]	
(N = 71)	26.9%		2.1%		45.7%		2.2%		21.3%		1.7%		100.0%		
**Unknown**	4,786	[5,423]	6,574	[6,133]	7,402	[18,928]	60	[232]	1,055	[2,059]	645	[833]	20,522	[19,336]	
(N = 15)	23.3%		32.0%		36.1%		0.3%		5.1%		3.1%		100.0%		
**Alive 1 year**	2,644	[3,138]	4,814	[5,372]	4,427	[10,847]	472	[812]	2,661	[4,477]	938	[1,038]	15,955	[12,202]	0.002
(N = 106)	16.6%		30.2%		27.7%		3.0%		16.7%		5.9%		100.0%		
**Dead 1 year**	3,677	[3,300]	1,066	[2,961]	4,301	[11,183]	293	[661]	3,418	[5,237]	237	[409]	12,991	[13,129]	
(N = 66)	28.3%		8.2%		33.1%		2.3%		26.3%		1.8%		100.0%		
**Total**	3,040	[3,231]	3,376	[4,939]	4,378	[10,945]	403	[761]	2,951	[4,782]	669	[918]	14,818	[12,611]	
(N = 172)	20.5%		22.8%		29.5%		2.7%		19.9%		4.5%		100.0%		
**SCLC** (mean, [sd], %)															
**Male**	2,708	[2,350]	-		4,646	[10,164]	878	[952]	2,747	[3,627]	694	[791]	11,674	[11,089]	0.078
(N = 23)	23.2%				39.8%		7.5%		23.5%		5.9%		100.0%		
**Female**	3,041	[1,905]	-		3,412	[2,889]	814	[912]	9,915	[8,846]	1,345	[1,272]	18,527	[9,054]	
(N = 3)	16.4%				18.4%		4.4%		53.5%		7.3%		100.0%		
**<65 years**	2,159	[1,993]	-		7,361	[13,510]	1,114	[981]	5,580	[6,000]	1,080	[930]	17,294	[14,093]	0.006
(N = 12)	12.5%				42.6%		6.4%		32.3%		6.2%		100.0%		
**≥65 years**	3,251	[2,443]	-		2,055	[2,824]	661	[864]	1,855	[2,726]	503	[706]	8,325	[4,718]	
(N = 14)	39.1%				24.7%		7.9%		22.3%		6.0%		100.0%		
**Limited disease**	1,689	[1,757]	-		4,851	[3,380]	1,671	[1,187]	6,133	[7,057]	1,074	[867]	15,418	[10,105]	0.140
(N = 7)	11.0%				31.5%		10.8%		39.8%		7.0%		100.0%		
**Extensive disease**	2,813	[2,175]	-		5,192	[11,989]	683	[624]	3,046	[3,672]	748	[888]	12,482	[12,007]	
(N = 16)	22.5%				41.6%		5.5%		24.4%		6.0%		100.0%		
**Unknown**	4,861	[3,018]	-		22	[39]	-		422	[731]	175	[208]	5,481	[2,378]	
(N = 3)	88.7%				0.4%				7.7%		3.2%		100.0%		
**Alive 1 year**	2,388	[2,047]	-		2,976	[2,784]	1,436	[1,093]	1,431	[2,652]	1,086	[1,008]	9,316	[3,443]	0.597
(N = 9)	25.6%				31.9%		15.4%		15.4%		11.7%		100.0%		
**Dead 1 year**	2,937	[2,420]	-		5,313	[11,724]	571	[692]	4,709	[5,378]	602	[735]	14,131	[13,140]	
(N = 17)	20.8%				37.6%		4.0%		33.3%		4.3%		100.0%		
**Total**	2,747	[2,272]	-		4,504	[9,578]	870	[930]	3,574	[4,826]	769	[852]	12,465	[10,943]	
(N = 26)	22.0%				36.1%		7.0%		28.7%		6.2%		100.0%		
**NCC** (mean, [sd], %)															
**Male**	3,901	[3,538]	1,100	[3,063]	183	[533]	36	[136]	4,768	[16,839]	129	[254]	10,116	[18,410]	0.645
(N = 25)	38.6%		10.9%		1.8%		0.4%		47.1%		1.3%		100.0%		
**Female**	3,802	[2,945]	871	[2,612]	9,586	[17,920]	427	[1,282]	2,597	[4,336]	447	[604]	17,729	[23,745]	
(N = 9)	21.4%		4.9%		54.1%		2.4%		14.6%		2.5%		100.0%		
**<65 years**	2,961	[3,609]	3,085	[4,261]	10,040	[19,035]	513	[1,350]	14,563	[28,241]	293	[476]	31,455	[34,191]	0.009
(N = 8)	9.4%		9.8%		31.9%		1.6%		46.3%		0.9%		100.0%		
**≥65 years**	4,156	[3,286]	410	[2,091]	405	[1,504]	25	[126]	1,002	[3,338]	189	[373]	6,186	[6,128]	
(N = 26)	67.2%		6.6%		6.5%		0.4%		16.2%		3.1%		100.0%		
**Stage I**	1,098	[998]	8,422	[0]	-		-		41,714	[58,992]	350	[496]	51,584	[59,495]	0.231
(N = 2)	2.1%		16.3%						80.9%		0.7%		100.0%		
**Stage II**	-		-		-		-		-		-		-		
(N = 0)															
**Stage III**	2,031	[2,498]	-		1,850	[3,700]	-		1,469	[1,300]	470	[550]	5,819	[6,680]	
(N = 4)	34.9%				31.8%				25.2%		8.1%		100.0%		
**Stage IV**	3,659	[2,869]	490	[1,959]	5,069	[13,981]	297	[962]	2,029	[4,090]	186	[436]	11,729	[18,728]	
(N = 16)	31.2%		4.2%		43.2%		2.5%		17.3%		1.6%		100.0%		
**Unknown**	5,239	[3,984]	888	[3,077]	195	[674]	-		1,733	[4,840]	142	[261]	8,197	[7,585]	
(N = 12)	64.1%		10.8%		2.4%				21.1%		1.7%		100.0%		
**Alive 1 year**	3,493	[2,978]	1,860	[3,735]	2,088	[6,677]	216	[881]	5,861	[19,080]	333	[478]	13,851	[22,728]	0.242
(N = 19)	25.2%		13.4%		15.1%		1.6%		42.3%		2.4%		100.0%		
**Dead 1 year**	4,358	[3,816]	-		3,411	[12,939]	43	[166]	2,080	[4,734]	62	[171]	9,954	[16,042]	
(N = 15)	43.8%				34.3%		0.4%		20.9%		0.6%		100.0%		
**Total**	3,875	[3,348]	1,039	[2,914]	2,672	[9,787]	140	[666]	4,193	[14,550]	213	[394]	12,132	[19,869]	
(N = 34)	31.9%		8.6%		22.0%		1.2%		34.6%		1.8%		100.0%		

NSCLC: non-small cell lung cancer.

SCLC: small cell lung cancer.

NCC: no cytohistologic confirmation.

sd: standard deviation.

In SCLC patients, the mean cost per patient with limited disease was 15,418 Euros, whereas the mean cost per patient with extensive disease was 12,482 Euros. The main cost components in the global mean cost per patient in SCLC were chemotherapy (36.1%) and other inpatient costs (28.7%).

Whereas large qualitative differences were observed between stages with regard to the aggregrate cost of treatment, the Kruskall-Wallis test did not show any statistically significant cost differences between stages in the mean cost per patient in the NSCLC, SCLC and NCC groups (p-value = 0.289, 0.140 and 0.231, respectively). Mann–Whitney tests showed statistically significant cost differences between patients who did or did not survive at one year from diagnosis in NSCLC cases (p-value = 0.002) and between age groups in SCLC and NCC patients (p-value = 0.006 and 0.009 respectively).

Table 5 shows the mean cost of surgical, chemotherapy and radiotherapy treatments by histology and stage at diagnosis. In NSCLC, the mean cost of surgical interventions ranged from 7,621 Euros in Stage IV to 10,228 Euros in Stage III. The mean cost of chemotherapy treatment ranged from 1,949 Euros in Stage I to 7,179 Euros in Stage IV. The mean cost of radiotherapy ranged from 663 Euros in Stage IV to 1,308 Euros in Stage II. In SCLC patients, the global mean cost of chemotherapy and radiotherapy treatments were 3,778 Euros and 943 Euros, respectively. In NCC patients, the global mean cost of surgical interventions, and chemotherapy and radiotherapy treatments were assessed at 8,835 Euros, 6,988 Euros and 791 Euros, respectively. Regardless of the histology, high variability was observed in the mean cost of chemotherapy treatments.

**Table 5:** **Cost of surgery, chemotherapy and radiotherapy treatments (Euros, 2008)**

	**Surgical interventions**			**Chemotherapy treatments**			**Radiotherapy treatments**		
	**N**	**Mean cost (Euros) [sd]**		**N**	**Mean cost (Euros) [sd]**		**N**	**Mean cost (Euros) [sd]**	
**NSCLC**									
Stage I	33	9,566	[1,486]	14	1,949	[2,193]	10	1,275	[901]
Stage II	8	10,177	[1,411]	6	4,604	[4,772]	5	1,308	[692]
Stage III	7	10,228	[1,608]	34	2,911	[5,255]	20	1,298	[907]
Stage IV	3	7,621	[2,707]	68	7,179	[12,465]	35	663	[683]
Unknown	10	9,862	[1,419]	7	15,861	[23,014]	1	900	-
**Total**	**61**	**9,677**	**[1,585]**	**129**	**5,838**	**[11,185]**	**71**	**977**	**[825]**
**SCLC**									
Limited disease	-	-	-	11	3,087	[2,732]	12	975	[733]
Extensive disease	-	-	-	19	4,372	[11,056]	12	911	[430]
Unknown	-	-	-	1	67	-	0	-	-
**Total**	**-**	**-**	**-**	**31**	**3,778**	**[8,757]**	**24**	**943**	**[589]**
**NCC**									
Stage I	2	8,422	[0]	-	-	-	-	-	-
Stage II	-	-	-	-	-	-	-	-	-
Stage III	-	-	-	3	2,467	[3,378]	-	-	-
Stage IV	1	7,835	-	9	9,012	[11,535]	6	791	[607]
Unknown	1	10,660	-	1	2,335	-	-	-	-
**Total**	**4**	**8,835**	**[1,248]**	**13**	**6,988**	**[10,030]**	**6**	**791**	**[607]**

NSCLC: non-small cell lung cancer.

SCLC: small cell lung cancer.

NCC: no cytohistologic confirmation.

sd: standard deviation.

## Discussion

In this multicentre study, we assessed hospital costs of lung cancer diagnosis and treatment in 232 patients from 9 hospitals in Catalonia. There were 53 public hospitals, of which only 10 had a thoracic surgery unit. The 9 hospitals selected from this study were taken from this group. Therefore, this sample selection was considered to be representative of teaching surgical hospitals in Catalonia.

The results of this study show that there is no association between the mean cost per patient and the stage of the disease. Indeed, there were no statistical significant differences in the mean cost per patient between stages.

In NSCLC, surgery, diagnosis, chemotherapy and other inpatient care were the main components of the mean cost per patient. The relative weight of chemotherapy was not as high as expected because of the type of drug schemes used at that time. While the costs of Stages II and IV were quite similar, the relative weight of surgery and chemotherapy cost was higher in Stage II. As expected, and in accordance with clinical guidelines [17], advanced disease stages were associated with a decrease in the relative weight of surgery, but an increase in chemotherapy costs. This pattern was also observed in the mean costs of chemotherapy treatments received. In the final balance, these different costs may cancel each other out, explaining why no association was observed between mean cost per patient and the stage of the disease. For SCLC patients, chemotherapy was one of the main components of the mean cost per patient.

In both NSCLC and SCLC, the cost of other inpatient care was relatively important in terms of global mean cost per patient at all stages. This might be due to the comorbidity associated with these tumours.

In NSCLC cases, a statistical difference between the mean cost of patients surviving one year from diagnosis and those who died was observed, so among other reasons, cost differences might also be explained by their different lengths of survival. In SCLC patients, this difference was also observed, although it was not statistically significant.

In SCLC and NCC patients there were differences in the mean cost per patient between age groups, with older groups incurring more costs. This suggests that these patients had a higher comorbidity, perhaps due to their more advanced age.

In the last ten years, various studies have estimated the cost of lung cancer treatment, with different objectives, methodologies and health contexts. Most of these studies have analysed cost components, but their findings are not directly comparable because resource use is not classified into the same cost categories as in our study. However, using similar methodology, in the study carried out by Kang et al. [13], the relative weight of chemotherapy costs in the total mean cost per patient in NSCLC is similar to our findings, ranging from 5% in Stage I to 33% in Stage IV. However, in SCLC patients their results are lower than ours. Dedes et al. [4] did not distinguish between NSCLC and SCLC, and they found that the largest cost item corresponded to hospitalisation (71% of total cost). They also found that chemotherapy was responsible for 14% of the total cost.

Only a few studies have analysed the hospital cost of lung cancer treatment by stage at diagnosis. In the study carried out by Fleming et al. [12], the researchers found that the stage at diagnosis had a significant influence on diagnostic, treatment, and total hospital costs. Costs were lower when disease presentation was at an advanced stage, reflecting the shorter survival and lower tolerance to aggressive treatment among these patients, as well as the more limited treatment options available to them. On the other hand, increasing costs in advanced stages of the disease have been observed in other studies [13].

Our findings on the influence of the stage at diagnosis in mean cost per patient are consistent with those presented by Demeter et al. [3], where there was no clear correlation between mean cost per patient and stage at diagnosis.

We were not able to identify any study similar to ours in Spain. The study carried out by Isla et al. [5] analysed treatment patterns, use of resources and costs of advanced NSCLC patients and was based on consensus recommendations of different chemotherapy schemes. The results reported are much higher than in our study because they are not based on clinical practice; rather, they are theoretical costs of chemotherapy in advanced stage and do not take into account the variability of clinical practice.

Another study carried out by Arca et al. Y analysed the costs of lung cancer diagnosis. They found that the mean cost for diagnosing NSCLC was 5,070 Euros, while the cost of diagnosing SCLC was 3,692 Euros. These diagnostic costs were significantly higher than our findings, but the study was limited to the cases diagnosed in one teaching hospital in the city of Ourense, so the differences may reflect specific diagnostic patterns of that hospital, while our study reflects the multihospital cohort made up of all public hospitals in Catalonia.

The cost of lung cancer treatment presents an important variability within each stage (mainly in SCLC) due to the limited number of patients included in the analysis. Given the low incidence of SCLC, which is only around 15% of the total incident cases of lung cancer, the low number of SCLC cases included in our study was largely unavoidable.

One limitation of this study is that only teaching hospitals were included, and therefore a larger number of patients requiring complex treatment would be expected. However, we limited our study to teaching hospitals because of the availability of thoracic surgery units in these centres. The estimation of surgery cost and the relative weight of surgery in mean total cost was one of the objectives of this study. In this sense, these hospitals were included in order to take into account the overall treatment of lung cancer. Nonetheless, this sample selection was considered to be representative of the surgical hospitals. Moreover, these type of hospitals have detailed information on resource use available through electronic records.

Although our study covers a period of three years of follow-up after diagnosis, it doesn’t provide costing information by time period. It is worth mentioning that this limitation is common to all studies conducted to date given the difficulty in obtaining the information needed to analyse these parameters. Therefore, further studies are required in order to investigate cost information by time period.

## Conclusions

This study provides the costs of lung cancer treatment as calculated from patient file reviews. Chemotherapy and surgery were the most important components of costs. As far as we know, ours is the first study that provides hospital costs of lung cancer treatment based on a review of patient files from different hospitals in Spain. This cost analysis is a baseline study that will provide a useful source of information for future studies on cost-effectiveness and on the budget impact of different therapeutic innovations in Spain.
